# Hidden hearing loss in patients with Charcot-Marie-Tooth disease type 1A

**DOI:** 10.1038/s41598-018-28501-y

**Published:** 2018-07-09

**Authors:** Ji Eun Choi, Jin Myoung Seok, Jungmin Ahn, Yoon Sang Ji, Kyung Myun Lee, Sung Hwa Hong, Byung-Ok Choi, Il Joon Moon

**Affiliations:** 1 Department of Otorhinolaryngology - Head and Neck Surgery, Dankook University Hospital, Cheonan, Republic of Korea; 2 Department of Neurology, Soonchunhyang University Cheonan Hospital, Soonchunhyang University College of Medicine, Cheonan, Korea; 3 Department of Otorhinolaryngology - Head and Neck Surgery, Samsung Medical Center, Sungkyunkwan University School of Medicine, Seoul, Republic of Korea; 4 Hearing Research Laboratory, Samsung Medical Center, Changwon, Republic of Korea; 5 School of Humanities and Social Sciences, KAIST, Changwon, Republic of Korea; 6 Department of Otorhinolaryngology - Head and Neck Surgery, Samsung Changwon Hospital, Sungkyunkwan University School of Medicine, Changwon, Republic of Korea; 7 Department of Neurology, Samsung Medical Center, Sungkyunkwan University School of Medicine, Seoul, Republic of Korea

## Abstract

The aim of this study was to investigate hidden hearing loss in patients with Charcot-Marie-Tooth disease type 1 A (CMT1A), a common inherited demyelinating neuropathy. By using pure-tone audiometry, 43 patients with CMT1A and 60 healthy controls with normal sound detection abilities were enrolled. Speech perception in quiet and noisy backgrounds, spectral ripple discrimination (SRD), and temporal modulation detection (TMD) were measured. Although CMT1A patients and healthy controls had similar pure-tone thresholds and speech perception scores in a quiet background, CMT1A patients had significantly (*p* < 0.05) decreased speech perception ability in a noisy background compared to controls. CMT1A patients showed significantly decreased temporal and spectral resolution (both *p* < 0.05). Also, auditory temporal processing of CMT1A patients was correlated with speech perception in a noisy background (r = 0.447, *p* < 0.01) and median motor conduction velocity (r = 0.335, *p* < 0.05). Therefore, we assumed that demyelination of auditory nerve in CMT1A causes defective cochlear neurotransmission, which reduces temporal resolution and speech perception in a noisy background. Because the temporal resolution test was well correlated with the degree of demyelination in auditory and peripheral motor nerves, temporal resolution testing could be performed as an additional marker for CMT1A.

## Introduction

Charcot-Marie-Tooth (CMT) disease, the most common hereditary peripheral neuropathy, affects 1 in 2500 people^1^. CMT type 1 A (CMT1A) is the predominant subtype, accounting for an estimated 50% of CMT cases. CMT1A is a demyelinating peripheral neuropathy characterized by distal muscle weakness, sensory loss, areflexia, and slow motor and sensory nerve conduction velocities^2–4^. CMT1A is associated with chromosomal duplication of 17p12 including peripheral myelin protein 22 (PMP22) gene^2^.

Because the cochlear nerve is a peripheral nerve, CMT patients can exhibit sensory peripheral nerve deficit. Hearing impairment is a relatively common symptom of CMT1A^3^. Hearing is usually assessed in the clinic by using pure-tone audiometry which measures the smallest detectable levels of pure tones from 0.125 to 8 kHz. However, audiograms do not predict selective neural loss which does not affect sensitivity to weak sounds^5^. Thus, CMT1A patients could have normal audiograms even though they have difficulty hearing in their daily lives.

Hidden hearing loss is recently described as auditory neuropathy believed to contribute to speech discrimination and intelligibility deficits in people with normal audiograms^6,7^. The relationship between auditory deficit and disease progression of CMT1A remains elusive. The objective of this study was to evaluate hidden hearing loss and determine diagnostic values of new auditory tests by comparing auditory measures and disease progression in CMT1A patients.

## Results

### Basic characteristics of the study population

Ages did not differ significantly between the CMT1A group (mean: 32.4 ± 13.3 years, ranges: 14–62 years) and the control group (mean: 30.2 ± 12.8 years, ranges: 14–65 years) (*p* > 0.05, two-tailed t-test). Percentage of females did not differ significantly between the CMT1A group (53.5%) and the control group (51.7%) either (*p* > 0.05, two-tailed t-test). The side of tested ears did not differ significantly between the two groups either (CMT1A group: 31 right ears and 12 left ears, control group: 40 right ears and 20 left ears, *p* > 0.05, Chi- square test). Clinical information on CMT1A patients is shown in Table 1. Most CMT1A patients (40 of 43 patients) had mild functional disability scale (FDS, ≤ 3). Of these, 23 had mild CMT neuropathy score (CMTNS, ≤ 10) and 17 had moderate (10 < CMTNS ≤ 21) CMTNS. No CMT1A patients had prolonged click auditory brainstem response (ABR) latencies compared to normative data^8^. Also, no CMT1A patients showed absent distortion product otoacoustic emission (DPOAE). Motor nerve conduction velocities (MNCV) of the median nerve in all CMT1A patients were decreased (ranging from 12 to 38 m/s) compared to normative data (normal value ≥ 50.5 m/s)^9^. However, compound muscle action potential (CMAP) of the median nerve was decreased only in 7 CMT1A patients compared to normative data (normal value ≥ 6 mV).

**Table 1:** Clinical and neurological characteristics of patients with Charcot-Marie-Tooth disease type 1 A (CMT1A).

No	Genotype	Sex	Age at exam (years)	Age at onset (years)	FDS^a^	CMT NS^b^	DPOAE	Latency wave (ms)			Inter-peak			Median nerve MNCV^c^	Median nerve
								I	III	V	I–III	III–V	I–V		CMAP^d^
1	CMT1A	M	42	7	6	27	ND	ND	ND	ND	ND	ND	ND	Abs	Abs
2	CMT1A	F	27	18	1	9	present	1.92	3.86	5.78	1.94	1.92	3.86	19	11.2
3	CMT1A	F	52	48	2	13	present	1.82	3.90	5.82	2.08	1.92	4.00	22	9.3
4	CMT1A	F	24	10	3	14	present	1.80	3.92	5.66	2.12	1.74	3.86	18	6.2
5	CMT1A	F	50	32	3	19	present	1.92	3.96	5.96	2.04	2.00	4.04	17	1.1
6	CMT1A	F	43	33	3	16	present	2.00	3.80	5.70	1.80	1.90	3.70	20	6.8
7	CMT1A	F	23	12	1	6	present	1.68	3.88	5.66	2.20	1.78	3.98	19	14.5
8	CMT1A	F	48	43	1	6	present	1.67	3.70	5.62	2.03	1.92	3.95	29	8.5
9	CMT1A	M	26	24	0	5	present	1.46	3.60	5.64	2.14	2.04	4.18	31	11.9
10	CMT1A	M	39	35	1	7	present	1.72	3.96	5.74	2.24	1.78	4.02	23	8.5
11	CMT1A	F	17	1	4	24	present	1.60	3.82	5.74	2.22	1.92	4.14	12	3.9
12	CMT1A	M	51	42	1	11	present	2.06	4.22	6.14	2.16	1.92	4.08	23	8.5
13	CMT1A	M	45	44	0	5	present	1.62	3.86	5.82	2.24	1.96	4.20	26	10.4
14	CMT1A	F	19	11	1	8	present	1.54	3.34	5.42	1.80	2.08	3.88	23	14.5
15	CMT1A	F	43	33	2	11	present	2.32	4.26	5.96	1.94	1.70	3.64	16	6.2
16	CMT1A	F	19	6	1	5	present	1.56	3.66	5.84	2.10	2.18	4.28	29	11.1
17	CMT1A	F	22	10	2	7	present	1.56	3.62	5.58	2.06	1.96	4.02	19	10.6
18	CMT1A	M	23	12	1	7	present	1.64	4.06	5.92	2.42	1.86	4.28	20	19
19	CMT1A	M	19	18	0	5	present	1.48	3.66	5.76	2.18	2.10	4.28	26	9.6
20	CMT1A	F	14	8	1	6	present	1.64	3.56	5.32	1.92	1.76	3.68	21	9.0
21	CMT1A	M	47	42	1	9	present	1.78	3.98	6.06	2.20	2.08	4.28	23	11.5
22	CMT1A	F	62	50	2	13	present	1.60	3.80	5.52	2.20	1.72	3.92	16	1.1
23	CMT1A	M	16	8	1	2	present	1.78	3.72	5.80	1.94	2.08	4.02	20	14.4
24	CMT1A	M	19	12	1	6	present	1.52	3.72	5.62	2.20	1.90	4.10	17	12.4
25	CMT1A	M	19	12	2	13	present	1.90	3.98	5.70	2.08	1.72	3.80	22	8.2
26	CMT1A	F	14	13	1	9	present	1.74	3.74	5.76	2.00	2.02	4.02	22	15.6
27	CMT1A	F	43	42	2	9	present	CNT	4.02	5.78	CNT	1.76	CNT	24	4.9
28	CMT1A	F	39	8	3	20	present	CNT	4.18	5.92	CNT	1.74	CNT	18	3.1
29	CMT1A	M	30	3	3	19	present	1.46	3.56	5.32	2.10	1.76	3.86	18	14.2
30	CMT1A	M	42	8	4	23	present	1.74	3.86	5.98	2.12	2.12	4.24	14	3.2
31	CMT1A	F	46	18	3	16	present	1.82	3.92	5.68	2.10	1.76	3.86	29	13.0
32	CMT1A	M	17	15	0	4	present	1.44	3.72	5.54	2.28	1.82	4.10	33	19.7
33	CMT1A	F	45	8	2	14	present	1.72	3.84	5.68	2.12	1.84	3.96	24	9.8
34	CMT1A	M	26	8	2	16	present	1.86	3.92	5.90	2.06	1.98	4.04	16	8.5
35	CMT1A	F	15	12	0	4	present	1.58	3.80	5.56	2.22	1.76	3.98	38	18.0
36	CMT1A	F	16	11	0	5	present	1.70	3.92	5.82	2.22	1.90	4.12	33	12.4
37	CMT1A	M	23	13	1	8	present	1.66	4.00	5.90	2.34	1.90	4.24	18	10.9
38	CMT1A	M	62	28	3	15	present	1.78	3.78	5.72	2.00	1.94	3.94	20	7.5
39	CMT1A	M	38	20	3	15	present	1.60	3.78	5.80	2.18	2.02	4.20	20	9.0
40	CMT1A	F	40	28	2	11	present	1.74	3.80	5.74	2.06	1.94	4.00	21	6.0
41	CMT1A	F	48	41	1	7	present	1.70	3.64	5.52	1.94	1.88	3.82	22	9.1
42	CMT1A	M	42	5	3	18	present	1.74	3.82	5.86	2.08	2.04	4.12	21	5.8
43	CMT1A	M	26	11	1	9	present	1.74	3.70	5.58	1.96	1.88	3.84	22	11.7

*Abbreviations: Abs* = *absent; F* = *female; M* = *male; ND* = *not done; CNT* = *cannot measured*.

^*a*^*FDS* = *functional disability scale*, *scale range 0–8*^19^*;* ^*b*^*CMTNS* = *Charcot-Marie-Tooth neuropathy score*, *score range 0–36;*^18^ ^*c*^*MNCV* = *motor nerve conduction velocity: normal median nerve MNCV ≥ 51 m/s;* ^*d*^*CMAP* = *compound muscle action potential: normal median nerve CMAP ≥ 6*.*0 mV*.

### Auditory outcomes and hearing disability

**Figure 1:**
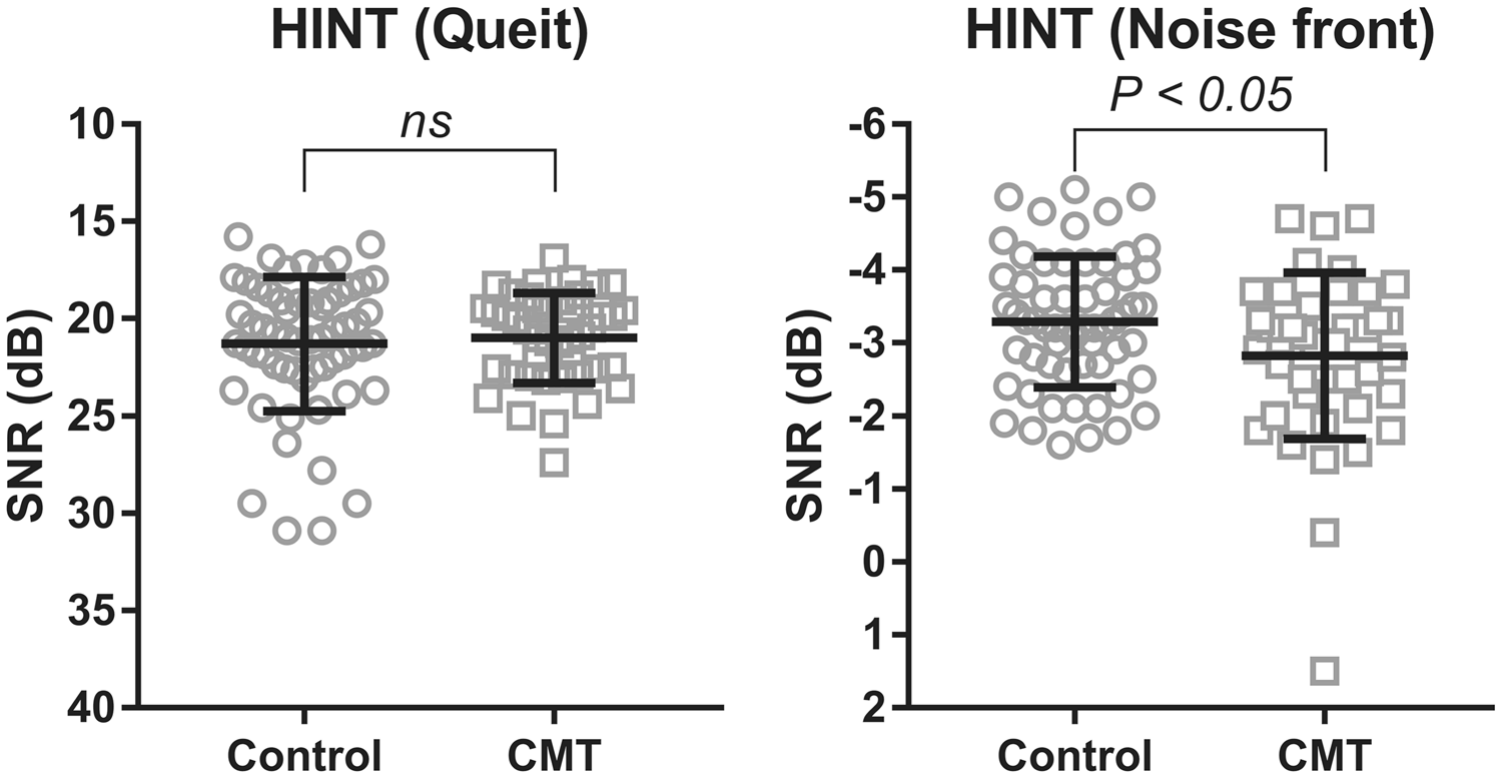
Sentence recognition scores in quiet and noisy backgrounds. The circle (○) and square (□) represent individual signal-to-noise ratio (SNR) thresholds in the control and CMT groups, respectively. A Korean version of Hearing in Noise Test (HINT) sentences was presented in quiet (A) and noisy backgrounds (B).

Auditory outcomes of tested ears and subjective hearing disabilities measured with APHAB and K-HHIE questionnaires for CMT1A patients and healthy controls are shown in Table 2 and Fig. 1. Pure tone thresholds at all frequencies (250, 500 Hz, 1, 2, 4, and 8 kHz), SRT, WRS, and subjective hearing disabilities were similar between the two groups. To evaluate hidden hearing loss in CMT1A patients with normal hearing, we tested sentence recognition in a noisy background using HINT and psychoacoustic tests (SRD and TMD tests). While CMT1A patients and healthy controls had similar speech perception scores in a quiet background (Fig. 1), the CMT1A group had significantly higher speech-to-noise ratio (SNR) thresholds than the control group (−2.83 ± 1.14 for CMT1A and −3.29 ± 0.90 for controls, *p* < 0.05, two-tailed t-test). Thus, CMT1A patients had more difficulty understanding speech in a noisy background compared to controls. Spectral and temporal resolutions for CMT1A patients were also significantly worse those than for healthy controls (Table 2). The mean SRD threshold was 3.67 ± 2.97 rpo for the CMT1A group and 4.58 ± 2.28 rpo for the control group (*p* < 0.05, two-tailed t-test). Mean TMD threshold was −14.73 ± 5.43 dB for the CMT1A group and −17.53 ± 2.78 dB for the control group (*p* < 0.05, Mann-Whitney test).

**Table 2:** Objective and subjective auditory outcomes for the CMT1A and control groups.

**Variable**	**Control (** ***n*** ** = 60)**	**CMT1A (** ***n*** ** = 43)**	***p*** **-value**
**Audiogram**			
Pure tone thresholds on tested ear (dB)			
250 Hz	9.50 ± 6.87	9.07 ± 6.84	ns
500 Hz	6.42 ± 5.83	7.44 ± 6.40	ns
1 kHz	7.08 ± 5.77	8.02 ± 5.89	ns
2 kHz	6.08 ± 6.04	5.70 ± 6.03	ns
4 kHz	8.83 ± 7.67	10.70 ± 8.76	ns
8 kHz	7.92 ± 9.27	8.95 ± 10.03	ns
SRT (dB)	5.77 ± 3.98	6.42 ± 5.60	ns
WRS (%)	98.47 ± 2.76	98.60 ± 2.64	ns
Psychoacoustic tests			
SRD threshold (rpo)	4.58 ± 2.28	3.67 ± 2.97	< 0.05
TMD threshold (dB)	−17.53 ± 2.78	−14.73 ± 5.43	< 0.05
Questionnaires			
K-HHIE	0.73 ± 1.52	1.53 ± 3.26	ns
APHAB			
Ease of communication	4.83 ± 5.08	6.58 ± 5.55	ns
Reverberation	7.25 ± 6.12	9.56 ± 7.66	ns
Background noise	7.62 ± 7.58	10.40 ± 8.32	ns
Aversion to sound	21.98 ± 18.02	27.77 ± 18.51	ns

*Abbreviations: SRT* = *speech reception threshold; WRS* = *word recognition score; HINT* = *Hearing In Noise Test; SNR* = *speech-noise ratio; SRD* = *spectral ripple discrimination; rpo* = *ripple per octave; TMD* = *temporal modulation detection; K-HHIE* = *Korean version of the Hearing Handicap Inventory for the Elderly; APHAB* = *Abbreviated Profile of Hearing Aid Benefit; ns* = *not significant*.

### Correlations between auditory measures for CMT1A patients

We conducted correlation analysis to investigate relationships among auditory neural conduction time (ABR wave I-V latency), ABR amplitude (ABR V/I ratio), spectral resolution (SRD), temporal resolution (TMD), and speech perception in quiet and noisy backgrounds (HINT). Findings for CMT1A patients with normal hearing are shown in Table 3. Temporal resolution was significantly correlated with speech perception in quiet (*r* = 0.411, *p* < 0.05) and noisy backgrounds (*r* = 0.447, *p* < 0.05). However, ABR inter-peak latency, ABR amplitude, or spectral resolution did not correlate with any other auditory measures. Thus, reduced temporal resolution could lead to impaired speech perceptions for CMT1A patients with normal hearing.

**Table 3:** Correlations between auditory measures in CMT1A patients.

Variables (control variable: age)		ABR (I–V) latency	ABR (V/I) amplitude	SRD thresholds	TMD thresholds	HINT (Quiet)
ABR (V/I) amplitude	*r*	−0.096				
	*p*	ns				
SRD thresholds	*r*	0.072	−0.068			
	*p*	ns	ns			
TMD thresholds	*r*	−0.159	0.042	−0.225		
	*p*	ns	ns	ns		
HINT (Quiet)	*r*	−0.279	−0.111	−0.093	**0**.**411**	
	*p*	ns	ns	ns	** < 0**.**05**	
HINT (Noise)	*r*	−0.239	−0.251	−0.05	**0**.**447**	**0**.**377**
	*p*	ns	ns	ns	** < 0**.**01**	** < 0**.**05**

*Abbreviations: ABR* = *auditory brainstem response; SRD* = *speech ripple discrimination; TMD* = *temporal modulation detection; HINT* = *hearing in noise test; ns* = *not significant*

*Bold numbers indicate significant difference at p* < *0*.*05*.

### Relationships between auditory measures and disease progression for CMT1A patients

Results of correlation analysis between auditory measures (ABRs, psychoacoustic tests, and HINT) and disease progression (age at onset, duration of disease, median nerve conduction study, FDS, and CMTNS) of CMT1A patients are shown in Table 4. Because auditory function would decrease with age, correlations between auditory measures and disease progression were conducted after controlling for age. In CMT1A patients, partial correlation analysis showed that TMD thresholds were significantly correlated with age at onset (*r* = −0.329, *p* < 0.05), disease duration (*r* = 0.329, *p* < 0.05), and median nerve conduction velocity (*r* = 0.335, *p* < 0.05). ABRs, spectral resolution, or speech perception did not correlate with any factors related to disease progression. This result indicated that temporal resolution was significantly decreased if CMT1A was developed earlier, the duration of CMT1A was longer, or the conduction velocity of median motor nerve was delayed.

**Table 4:** Partial correlation analysis between auditory and non-auditory measures in CMT1A.

Variables (control variable: age)		Age at onset	Disease duration	median MNCV	FDS	CMTNS
ABR (I–V) latency	*r*	−0.005	0.005	0.119	−0.285	−0.122
	*p*	ns	ns	ns	ns	ns
ABR (V/I) amplitude	*r*	0.047	−0.047	−0.256	0.176	0.217
	*p*	ns	ns	ns	ns	ns
SRD thresholds	*r*	−0.198	0.198	−0.132	−0.048	−0.047
	*p*	ns	ns	ns	ns	ns
TMD thresholds	*r*	**−0**.**329**	**0**.**329**	**0**.**335**	−0.011	0.065
	*p*	** < 0**.**05**	** < 0**.**05**	** < 0**.**05**	ns	ns
HINT (Quiet)	*r*	−0.144	0.144	0.079	0.182	0.016
	*p*	ns	ns	ns	ns	Ns
HINT (Noise)	*r*	0.064	−0.064	0.315	0.017	−0.073
	*p*	ns	ns	ns	ns	ns

*Abbreviations: ABR* = *auditory brainstem response; SRD* = *speech ripple discrimination; TMD* = *temporal modulation detection; HINT* = *hearing in noise test; MNCV* = *motor nerve conduction velocity; CMAP* = *median motor amplitude; FDS* = *functional disability scale; CMTNS* = *Charcot-Marie-Tooth neuropathy score; ns* = *not significant*

*Bold numbers indicate significant difference at p* < *0*.*05*.

## Discussion

This study evaluated hidden hearing loss of CMT1A patients and found that they had significantly reduced speech perception in a noisy background compared to controls (Fig. 1). Their difficulty understanding speech in a noisy background might be due to impaired temporal resolution (Table 3, *r* = 0.447, *p* < 0.01). Speech comprises dynamic spectral and temporal modulations that can change depending on utterance. Spectral and temporal modulation sensitivities are important factors affecting speech perception^10,11^. In our study, psychoacoustic tests revealed that CMT1A patients could not distinguish subtle differences in spectral shape (i.e., frequency) or rapid fluctuations in the envelope (i.e., event in time) compared to controls (Table 2).

The precise mechanism of hidden hearing loss in CMT1A is not fully understood yet. All CMT1A patients showed normal cochlear function based on DPOAE test (Table 1). Auditory nerve demyelination in CMT1A might have resulted in decreased speech perception in a noisy background. Auditory nerve demyelination could disrupt the neural insulator and cause slowed/inconsistent conduction of neural inputs^12^. Demyelination can affect the synchrony of neural discharge and increase temporal dispersion^13^. Hidden hearing loss often referred to as cochlear synaptopathy^5,14^ but, our findings indicate that cochlear neuropathy in CMT1A also regards as different type of hidden hearing loss. Temporal processing ability in CMT1A patients was significantly worse in our study, indicating that neural phase locking is impaired. Similarly, in previous studies, psychoacoustic experiments performed on patients with various auditory neuropathies have identified significant impairment in temporal processing^3,4,15–17^. Interestingly, CMT1A patients also showed impaired spectral resolution compared to controls (Table 2). Moore and Sek (1996) have suggested that temporal mechanisms might contribute to the detection of spectral modulation for carrier frequencies below 4 kHz with very low modulation rates^18^. Thus, impaired temporal cues might play an important role in discriminating a spectrally rippled stimulus in CMT1A patients.

The sensitivity of the auditory system to neural damage in patients with CMT1A indicates that hearing measurements can be used as clinical indicators of disease progression. Because both peripheral and central auditory physiology could change with age, we conducted a correlation analysis adjusted for age when evaluating the relationship between auditory measures and disease progression in patients with CMT1A (Table 4). Our results (Table 4) indicated that auditory temporal envelope processing was significantly correlated with age at onset (*r* = −0.329, *p* < 0.05), disease duration (*r* = 0.329, *p* < 0.05), and median MNCV (*r* = 0.335, *p* < 0.05). Therefore, hearing measurements can be used to track neural damage as the disease progresses. CMTNS has been used as a major outcome measure for CMT1A. However, CMTNS has limited sensitivity in CMT1A patients with mild clinical symptoms^19^. CMT1A is a slowly progressive neuropathy. Thus, detecting the disease progression and evaluating the efficacy of interventions are difficult^20^. Because temporal resolution test (TMD test) was associated with hidden hearing loss (reduced speech perception in a noisy background) and neural deficit (delayed median MNCV), temporal resolution test can be used to detect disease progression in CMT1A patients with mild clinical symptoms.

Although auditory function in children with CMT1A has been reported^3^, this study has several strengths. Our study had large number of participants with normal sound detection across all ages. Detailed psychoacoustic tests (SRD and TMD) were conducted to evaluate auditory processing associated with auditory neuropathy. We also analyzed auditory processing after controlling for age in participants with the same hearing configuration.

Results of this study showed that CMT1A patients had hidden hearing loss compared to healthy controls, although all participants had normal audiogram. Their difficulty understanding speech in a noisy background might be due to impaired ability to handle temporal envelope clues. We assumed that demyelination of auditory nerve cause defective cochlear neurotransmission and this cochlear neuropathy could be consider as different type of hidden hearing loss. Because auditory temporal envelope processing was related to median MNCV, temporal processing ability might be a suitable marker of disease progression. Therefore, temporal resolution testing could be performed in the clinical setting to detect hidden hearing loss and monitor the degree of neural damage. Future longitudinal studies are needed in this direction to assess the role of auditory measures as biomarker in CMT1A patients with mild to severe symptoms.

## Methods

All participants were recruited and tested in the Hearing Laboratory at Samsung Medical Center. Every participant provided written informed consent to participate in this study. The study protocol was approved by Samsung Medical Center Institutional Review Board (IRB No. 2015-04-075). This study was carried out in accordance with approved guidelines.

### Subjects

**Figure 2:**
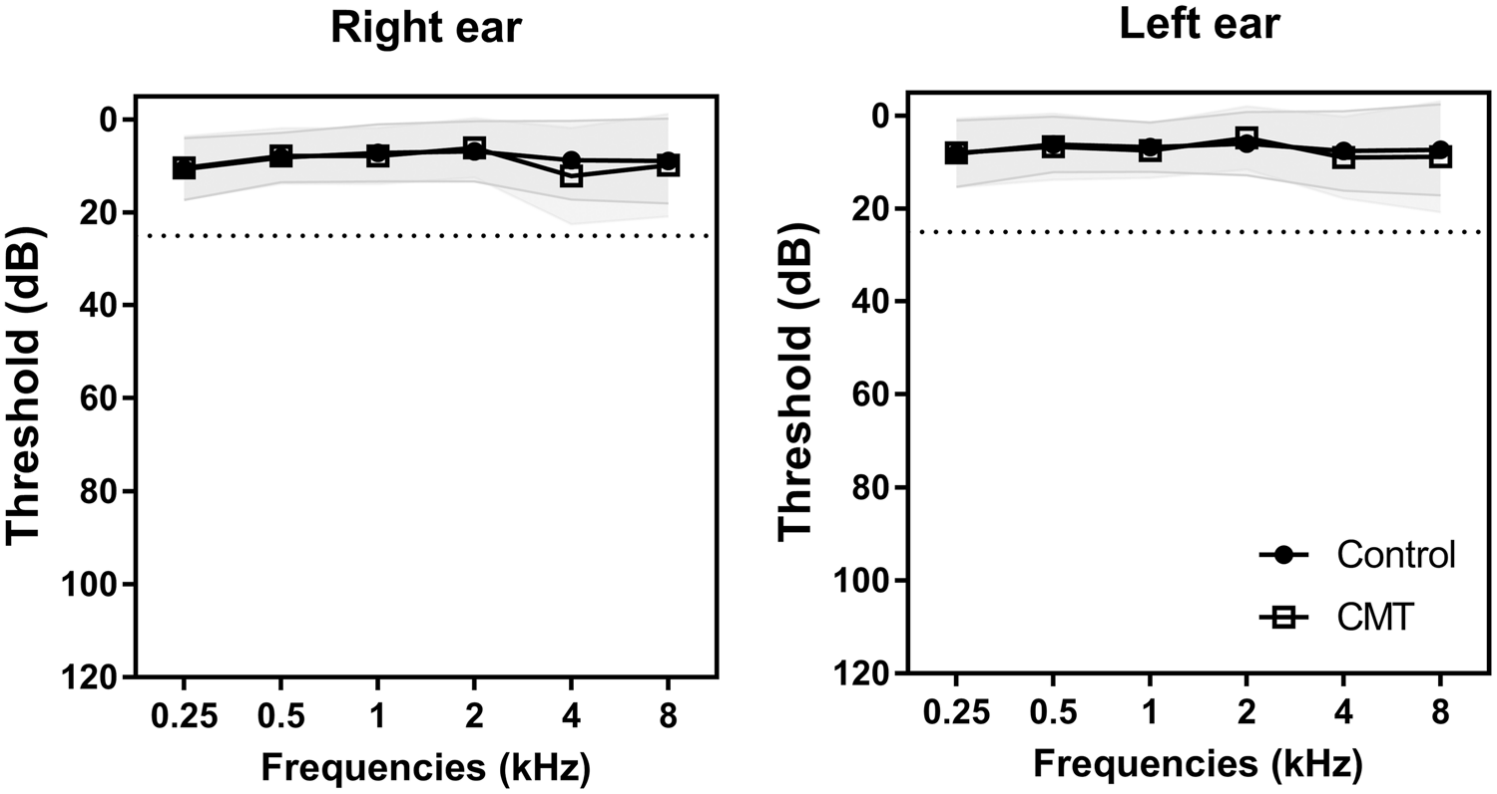
Audiograms for CMT1A patients and healthy controls. Average pure tone thresholds at each frequency (from 250 Hz to 8 kHz) are shown with circles (●) and squares (□) for the CMT1A group and the control group, respectively. All participants had an average hearing threshold of less than 25 dB at 0.5, 1, 2, and 4 kHz in both ears.

Forty-three CMT1A patients (20 males and 23 females) and 60 healthy controls (29 males and 31 females) were enrolled. On pure-tone audiometry, all study populations had an average hearing threshold of less than 25 dB at 0.5, 1, 2, and 4 kHz in both ears. Pure-tone thresholds of both groups are shown in Fig. 2. All CMT1A patients had demyelinating peripheral neuropathies with confirmed PMP22 gene duplication^21^. Age at onset was determined by asking patients their age when symptoms first appeared. To determine physical disability, two scales were used: CMT neuropathy score (CMTNS)^22^ and functional disability scale (FDS)^23^.

### Electrophysiology

For neurophysiologic study, auditory brainstem responses (ABRs), distortion product otoacoustic emission (DPOAE), and nerve conduction of median nerves were recorded in CMT1A patients. The equipment used to record brainstem auditory evoked potentials was Nicolet Viking Select (Natus Medical Inc., USA). Click stimuli at 80 dB nHL were transmitted via inserted earphone. Absolute latency (stimulus to peak) of each peak (I, III, and V) and inter-peak latencies (I–III, I–V, and III–V) were measured. DPOAE was recorded using an ILOV6 OAE analyzer (Otodynamics Ltd., USA). Primary signals f1 and f2, with f2/f1 = 1.22, generated with test frequencies ranging from 1001 Hz to 6006 Hz with a frequency resolution of one DPOAE per octave was used. Two levels were chosen: L1 = 65 dB SPL, L2 = 55 dB SPL. Response parameters to consider DPOAE as present included DP amplitude and SNR. A peak at 2f1-f2 in the spectrum was accepted as a DPOAE if it was 3 dB above the noise floor. Nerve conduction studies were performed by placing surface electrodes on median nerves. Motor nerve conduction velocities (MNCVs) for median nerves were determined by stimulating at the elbow and wrist while recording compound muscle action potentials (CMAPs) over the abductor pollicis brevis muscle.

### Audiograms and speech recognition tests

Audiograms were used to assess pure tone threshold, speech perception threshold (SRT), and word recognition score (WRS). Pure-tone thresholds were obtained under headphones using pure-tones ranging from 250 Hz to 8 kHz. They were measured with ascending 5 dB method (modified Hughson-Westlake method)^24^ using two different audiometers (Orbiter 922, Madsen Electronics, Taastrup, Denmark and GSI-61, Grason-Stadler, USA). Word recognitions were measured at the most comfortable level. Sentence recognition tests were performed in quiet and noisy backgrounds with a Korean version of Hearing in Noise Test (HINT) sentences^25^.

### Hearing disability

Hearing disability was assessed with questionnaires such as the abbreviated profile of hearing aid benefit (APHAB)^26^ and hearing handicap inventory for the elderly (HHIE)^27^. Everyday listening and communication were investigated with the APHAB questionnaire. This 24-question metric explores four aspects of auditory function: communication difficulty, effect of background noise, effect of reverberation, and aversion to loud sounds. Effects of hearing impairment on emotional and social adjustment were investigated with the HHIE questionnaire, with higher scores for the questionnaire indicating greater discomfort related to hearing.

### Psychoacoustic tests

Psychoacoustic tests consisted of spectral ripple discrimination (SRD) and temporal modulation detection (TMD) tests. A custom-made MATLAB® (The Mathworks, Natick, MA, USA) graphical user interface was used to present acoustic stimuli to subjects for SRD and TMD tests. The stimuli were presented monoaurally through an insert earphone (ER-3A, Etymotic) at an average of 65 dBA.

### Spectral ripple discrimination (SRD)

SRD thresholds were determined with stimuli based on a previously established technique^28–30^. Three rippled noise tokens with a 30-dB peak-to-trough ratio, two with a standard ripple phase, and one with an inverted ripple phase were created from 2,555 tones. The spectral modulation starting phase of the full-wave-rectified sinusoidal spectral envelope was set to zero radian for standard ripple stimuli. The spectral modulation starting phase of the inverted spectral-ripple stimulus was set to π/2 radians. The bandwidth of the rippled spectrum was 100–5000 Hz. The stimulus duration was 500 ms. The order of presentation of the three tokens was randomized and the subject’s task was to select the “oddball” stimulus. To measure SRD thresholds, a three-interval, three-alternative forced choice (3-AFC) paradigm with an adaptive two-up, one-down procedure was used. Ripple density varied between 0.125 and 11.314 ripples per octave (rpo) in equal ratio steps of 1.414 in an adaptive manner with 13 reversals that converged to the 70.7% correct point^31^. A level attenuation of 1–8 dB (in 1 dB increments) was randomly selected for each interval in the three-interval task. The SRD threshold for each adaptive run was calculated as the mean of the last eight reversals. The SRD threshold for each subject was the mean of three adaptive runs. Higher spectral-ripple thresholds indicated better discrimination performance.

### Temporal modulation detection (TMD)

The TMD test was administered by using previously described procedures^11,30,32^. The stimulus duration was one second for both modulated and unmodulated signals. For modulated stimuli, sinusoidal amplitude modulation was applied to a wideband noise carrier. For unmodulated stimuli, continuous wide band noise was applied. A modulation frequency of 10 Hz was tested. Modulated and unmodulated signals were gated on and off with 10-ms linear ramps. They were concatenated without gap between signals. The TMD threshold was measured with a two-interval, two-alternative adaptive forced choice (2I, 2-AFC) paradigm. One interval consisted of modulated noise while the other consisted of unmodulated white noise. Subjects were asked to identify the interval that contained the modulated noise. A two-down, one-up adaptive procedure was used to measure TMD threshold, starting with a modulation depth of 100% and decreasing in 4-dB steps from the first four reversals and 2-dB steps for the next 10 reversals. For each testing run, the final 10 reversals were averaged to obtain the TMD threshold. TMD thresholds in decibels relative to 100% modulation (i.e., 20 ${{\rm{log}}}_{10}{m}_{i}$) were obtained, where ${{\rm{m}}}_{i}$ indicated the modulation index. The threshold for each subject was calculated as the mean of three testing runs. Lower modulation depths indicated better performance.

### Statistical analysis

Results were analyzed with SAS version 9.4 (SAS Institute Inc., Cary, NC, USA). Independent t-tests were performed to assess significant differences in auditory tests between CMT1A patients and control participants. Binary logistic regression analyses were performed to assess related auditory processing in CMT1A patients compared to controls. Each auditory measure was adjusted for age and sex. Correlation coefficient was calculated to investigate whether auditory measures were correlated among themselves or with age. Correlation coefficients between auditory and non-auditory measures were calculated after controlling for age.
